# Can Wild Ungulate Carcasses Provide Enough Biomass to Maintain Avian Scavenger Populations? An Empirical Assessment Using a Bio-Inspired Computational Model

**DOI:** 10.1371/journal.pone.0020248

**Published:** 2011-05-24

**Authors:** Antoni Margalida, Ma. Àngels Colomer, Delfí Sanuy

**Affiliations:** 1 Bearded Vulture Study and Protection Group, El Pont de Suert Lleida, Spain; 2 Division of Conservation Biology, Institute of Ecology and Evolution, University of Berne, Bern, Switzerland; 3 Department of Mathematics, University of Lleida, Lleida, Spain; 4 Department of Animal Production, University of Lleida, Lleida, Spain; University of Bern, Switzerland

## Abstract

**Background:**

The reduction in the amount of food available for European avian scavengers as a consequence of restrictive public health policies is a concern for managers and conservationists. Since 2002, the application of several sanitary regulations has limited the availability of feeding resources provided by domestic carcasses, but theoretical studies assessing whether the availability of food resources provided by wild ungulates are enough to cover energetic requirements are lacking.

**Methodology/Findings:**

We assessed food provided by a wild ungulate population in two areas of NE Spain inhabited by three vulture species and developed a P System computational model to assess the effects of the carrion resources provided on their population dynamics. We compared the real population trend with to a hypothetical scenario in which only food provided by wild ungulates was available. Simulation testing of the model suggests that wild ungulates constitute an important food resource in the Pyrenees and the vulture population inhabiting this area could grow if only the food provided by wild ungulates would be available. On the contrary, in the Pre-Pyrenees there is insufficient food to cover the energy requirements of avian scavenger guilds, declining sharply if biomass from domestic animals would not be available.

**Conclusions/Significance:**

Our results suggest that public health legislation can modify scavenger population trends if a large number of domestic ungulate carcasses disappear from the mountains. In this case, food provided by wild ungulates could be not enough and supplementary feeding could be necessary if other alternative food resources are not available (i.e. the reintroduction of wild ungulates), preferably in European Mediterranean scenarios sharing similar and socio-economic conditions where there are low densities of wild ungulates. Managers should anticipate the conservation actions required by assessing food availability and the possible scenarios in order to make the most suitable decisions.

## Introduction

The crises among avian scavengers in the Old World during the last few years of the 20^th^ century have revealed the sensitivity of vulture populations to the sudden appearance of non-natural mortality factors. In Asia and Africa, the massive decline in several species as a result of the ingestion of veterinary drugs has forced managers and policy-makers to seek urgent conservation measures [1]–[4]. European vultures have also been affected and, in recent years, the status of their populations has become precarious, with the appearance and increases of non-natural mortality factors that may have an impact on their dynamics. Recent studies of the appearance of veterinary drugs [5], [6], the illegal used of poisoned bait [7], [8], the increase in the number of wind farms and the corresponding death through collisions with wind turbines [9], [10] and lead poisoning as a result of the ingestion of prey that was killed using lead shot [11], [12] are some of the most serious concerns from a conservation point of view.

However, in addition to this, one of the limiting factors that has generated the greatest amount of debate in recent years, and may also the most worrying for the conservation of European vultures, is the impact that restrictive public health policies may have on European avian scavenger populations in general, and on Spanish populations in particular, as a result of the reduction in the amount of food available to these birds [13], [14]. During the first decade of the 21^st^ century, following the outbreak of bovine spongiform encephalopathy, in Europe most carcasses of domestic ungulates were removed, limiting food resources for avian scavengers [13]–[16]. The application of this public health legislation contradicted environmental conservation policies, preventing avian scavengers from obtaining the most important food resources (i.e. domestic ungulates), on which their diet has been based for centuries. Several warning signs, such as the decrease in productivity, the increase of attacks on livestock and the increase in mortality in young age classes, made it essential to change the legislation and also to increase the number of supplementary feeding sites as a partial solution [13].

Since domestic ungulates provide most of the carcasses available to scavengers [17], what would be the effect of their disappearance or reduction on scavenger populations? What would the dynamics of local scavenger populations be if they had to feed only on local wildlife? The answer to these questions could elucidate the carrying capacity of some areas for some scavengers in the absence of domestic ungulates with only the existence of wild ungulates. Although these are important questions for managers and policy-makers, up to now no study on this subject has focused on assessing whether food biomass provided by wild ungulate carcasses is sufficient to cover the energy requirements of an avian scavenger guild. The only related study, carried out recently, focused on the importance of hunting activities on food supplies for a Eurasian griffon vulture *Gyps fulvus* population in an area of northern Spain [18]. Taking into account that in recent decades the Spanish wild ungulate populations have experienced a notable increase in distribution area as well as in the number of individuals, the carcasses of these species are gradually becoming more available and may constitute an important food source for vertebrate scavengers [19]–[21]. Knowledge of feeding resources provided by wild ungulates can allow managers and policy-makers to anticipate conservation measures before any possible changes in public health legislation. Along these lines, bearing in mind that in Europe, 90% of the Eurasian griffon vulture, cinereous vulture *Aegypius monachus* and Egyptian vulture *Neophron percnopterus* populations, and over 62% of the bearded vulture *Gypaetus barbatus* population, are found in Spain, the conservation of these species in Europe depends to a large extent on the management carried out in this country.

Here, we take the advantage of the recent development of the P Systems computational models [22] as a potential and robust tool for modelling experimental data on avian scavenger and wild ungulate populations [23]–[24], in order to assess the effects of carrion resources on population dynamics. The difference in P systems with respect to other Multi-Agent Systems (MAS [25]) is basically the type of rules applied. Whereas in other MAS, the model conceptualization in terms of agents can be implemented with mathematical equations [26], in P systems this is not possible. In these models the agents are implemented by particular rules. This type of model facilitates the simultaneous study of a large number of species, which evolve in parallel and interact, something that cannot be achieved with the other models such as differential equations, viability models and multy-agent systems [26], [27], [28].

In this work, taking as a case study a large area of NE Spain inhabited by avian scavengers, and in which monitoring has been carried out intensively over the last two decades, we test the role of wild ungulates in vulture management and conservation and population growth. Our main goal is to model an ecosystem inhabited by three vulture species (the bearded, Egyptian and griffon vulture) to see if they are able to survive on nothing more than the food provided by a community of wild ungulates. More specifically our aims are: 1) to evaluate the importance of wild ungulates as a food resource for an avian scavenger guild in an intensively monitored zone; 2) to determine which species provided the most wild animal biomass and to analyse the importance of their management for population growth; and 3) to discuss whether artificial management such as the creation of supplementary feeding sites in a hypothetical trophic limitation scenario (i.e. scarce availability of food provided by livestock) resulting from public health policies can be necessary in the future.

## Results

### Food provided by wild ungulates in the Pyrenean region

**Figure 1:**
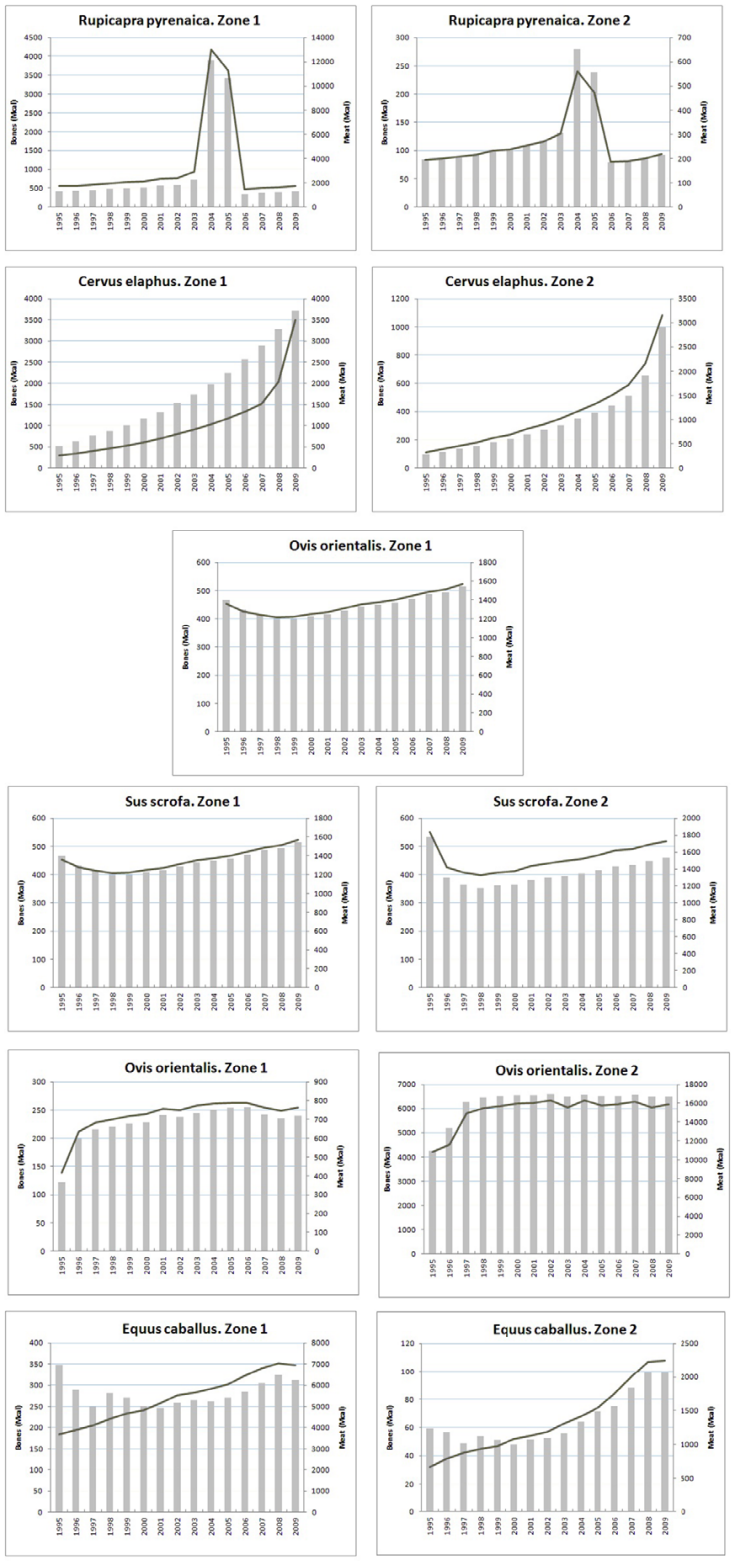
Annual variation in the biomass (megacalories) provided by wild ungulates in the Pyrenees (Zone 1) and Pre-Pyrenees (Zone 2). Bars: meat; line: bones.

**Figure 2:**
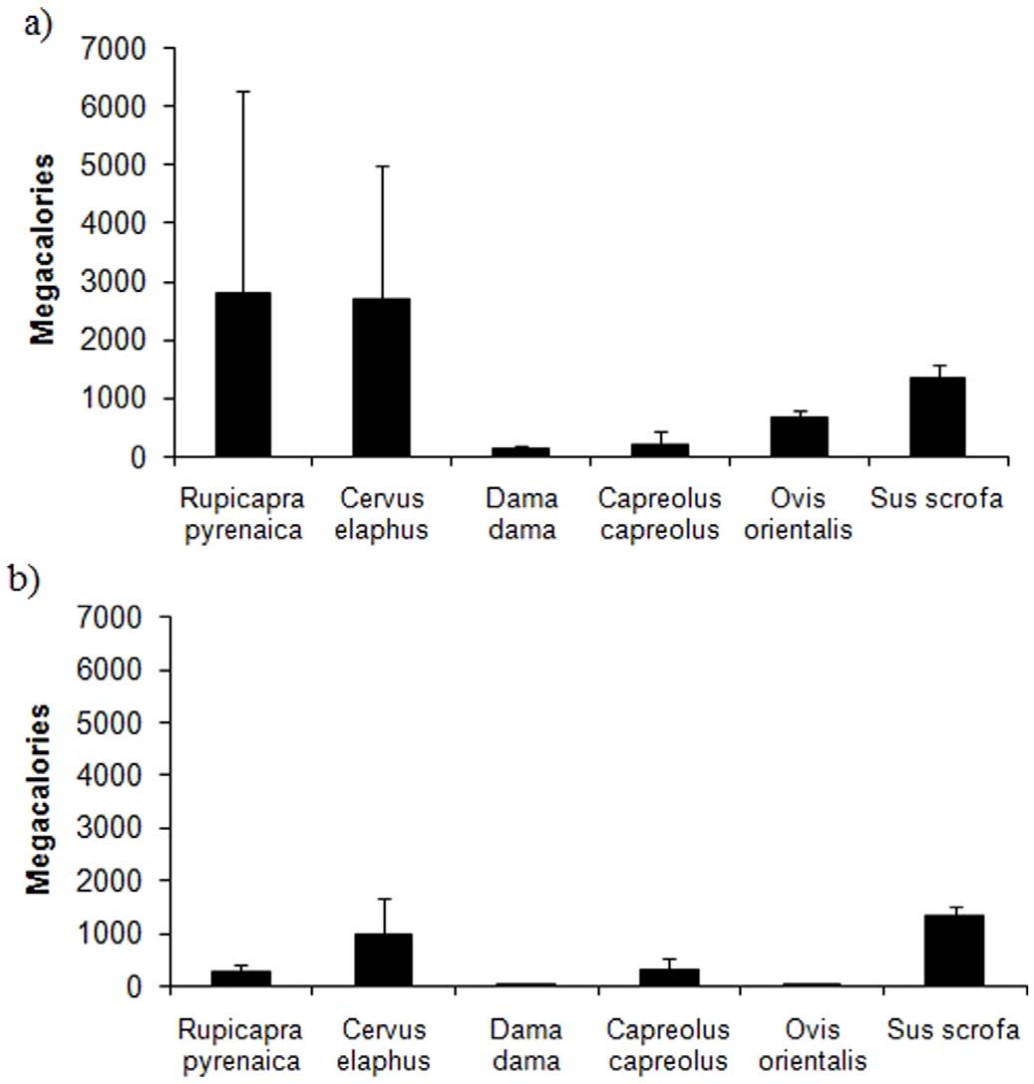
Average biomass (megacalories ±1 SD) provided (bones and meat) by the different wild ungulate species in the Pyrenees (a) and Pre-Pyrenees (b). The columns represent the average biomass provided during the 15 years simulated.

We found significant differences between the bone remains provided by the six ungulate species present in the Pyrenean region (*F* = 10.54, d.f. = 5, 84, *P*<0.0001), with the Pyrenean chamois *Rupicapra pyrenaica* and red deer *Cervus elaphus* being the species that provided significantly more animal biomass than the remaining species (Duncan test, *P*<0.05, Figures 1 and 2). In the case of Pyrenean chamois, note that in the period simulated corresponding to 2004–2005 the significant increase of food available is consequence of a disease (pestivirus).

With regard to meat remains, we found significant differences between species (*F* = 7.40, d.f. = 5, 84, *P*<0.0001) with the Pyrenean chamois and red deer again being the species that provided more meat animal biomass (Duncan test, *P*<0.05, Figures 1 and 2) when compared with the remaining species.

### Food provided by wild ungulates in the Pre-Pyrenean region

We found significant differences between the bone remains provided by the six ungulate species present (*F* = 53.36, d.f. = 5, 84, *P*<0.0001). The homogeneity test identified three groups, with the red deer and wild boar *Sus scrofa* being the species that provided most bone biomass (Duncan test, *P*<0.05, Figures 1 and 2) as compared to the rest. In the second group, the Pyrenean chamois also provided significantly more biomass than the remaining species (fallow deer *Dama dama*, mouflon and roe deer *Capreolus capreolus*), which comprised the third group, and were of negligible importance.

With regards the meat remains, again we found significant differences between species (*F* = 47.69, d.f. = 5, 84, *P*<0.0001) with the wild boar being the most commonly found species, separated from the rest (Duncan test, *P*<0.05). The red deer appeared in second place separated from the rest, and the Pyrenean chamois and roe deer were placed in a third group, separated from the fallow deer and mouflon *Ovis orientalis* (Duncan test, *P*<0.05, Figures 1 and 2).

### Differences in the biomass available: Pyrenees vs Pre-Pyrenees

We found significant differences in the average amount of bone biomass provided by wild ungulates in the Pyrenees *vs* the Pre-Pyrenees (*t* = 4.96, *P*<0.0001, Figure 2) with the average of the differences being 1,872 megacalories more in the Pyrenees than in the Pre-Pyrenees. As far as meat remains are concerned, once again in the Pyrenees food availability is significantly higher than in the Pre-Pyrenees (*t* = 4.34, *P*<0.0001), with the average of the differences being 6,835 megacalories more than in the Pre-Pyrenees.

### Population dynamics both with all biomass available, and with wild ungulates only

**Figure 3:**
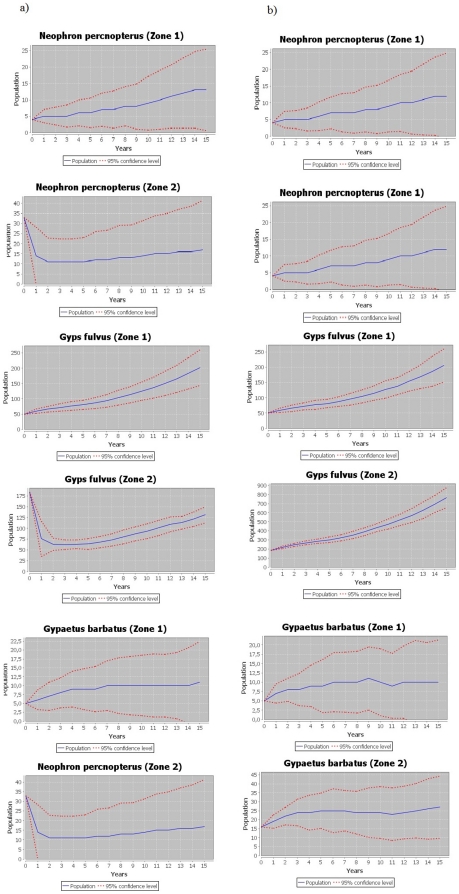
Hypothetical population trend and 95% confidence intervals of the three avian scavengers in the two subpopulations studied. a) represents a hypothetical scenario in which food obtained by the scavengers was only provided by wild ungulates) while b) represents the results obtained by the model under the real scenario during 1994–2009. The starting point (0) is the population of every species in the year 1994. Zone 1): Pyrenees; Zone 2): Pre-Pyrenees.

As shown in Figure 3, in the Pyrenees the increase in the guild of avian scavengers would not have been affected if they had only access to food provided by wild ungulates (Figure 3a). With regards to the population growth in the real scenario, no significant differences were found in any of the species considered (*P*>0.05 for all species, Figure 3a). The average growth of the bearded vulture population with wild ungulates only was 5±5%, for the Egyptian vulture 8±4% and for the griffon vulture 10±14%.

In contrast, in the Pre-Pyrenees, if biomass from domestic animals would not be available, populations of the three species decline sharply. In the cases of the griffon and Egyptian vulture, the populations would recover over time and as the number of wild prey species increases (Figure 3a). In the case of the bearded vulture, the decline caused by the elimination of biomass from domestic animals would be so great that the population would decrease to a level from which it will be hard for it to recover. Thus, in the Pre-Pyrenees, there are statistically significant differences, especially in the case of the bearded vulture (average growth with wild ungulates only: −5±6%, growth in the real scenario: 4±3%, *P* = 0.028) and the griffon vulture (with wild ungulates only: 0.03±9%, growth in the real scenario: 10±1.6%, *P* = 0.039), being marginally significant in the case of the Egyptian vulture (with wild ungulates only: −2±8%, growth in the real scenario: 6±0.8%, *P* = 0.061).

With respect the elasticity analyses of the demographic parameters, the population of the three avian scavengers proved to be especially sensitive to variations in k1 and to a lesser degree, in k2 (Table 1), both related with the breeding process.

**Table 1:** Results of the elasticity analyses of the demographic parameters of the griffon, Egyptian and bearded vulture population and separately for the two geographical areas (Zone 1: Pyrenees; Zone 2: Pre-Pyrenees).

		g4		m1		m2		k1		k2	
Bearded vulture	*Range values*	5	8	0.04	0.1	0.04	0.12	0.6	0.8	0.3	0.7
	*Zone 1*	−0.086	−0.057	0.000	−0.007	−0.105	−0.057	0.100	0.100	0.095	0.131
	*Zone 2*	−0.062	−0.056	−0.005	−0.010	−0.068	−0.055	0.120	0.120	0.098	0.085
	*Average*	−0.074	−0.057	−0.003	−0.009	−0.087	−0.056	0.110	0.110	0.097	0.108
	*Range values*	6	7	0.04	0.1	0.04	0.12	0.65	0.85	0.33	0.77
Egyptian vulture	*Zone 1*	−0.095	−0.056	−0.010	−0.005	−0.057	−0.051	0.119	0.071	0.090	0.081
	*Zone 2*	−0.047	−0.051	−0.003	−0.003	−0.007	−0.050	0.058	0.025	0.084	0.008
	*Average*	−0.071	−0.053	−0.006	−0.004	−0.032	−0.050	0.089	0.048	0.087	0.045
	*Range values*	6	7	0.04	0.1	0.04	0.12	0.6	0.8	0.3	0.7
Griffon vulture	*Zone 1*	−0.067	−0.055	−0.007	−0.007	−0.077	−0.051	0.106	0.132	0.094	0.118
	*Zone 2*	−0.074	−0.060	−0.005	−0.009	−0.050	−0.052	0.110	0.123	0.096	0.078
	*Average*	−0.071	−0.057	−0.006	−0.008	−0.063	−0.051	0.108	0.128	0.095	0.098

g4: age at which fertility begins; m1: natural mortality ratio in first years; m2: mortality ratio in adult animals; k1: proportion of pairs that can breed; k2: proportion of pairs with successful breeding.

## Discussion

These results constitute the first assessment of the effects that food provided by wild ungulates alone could have on the population dynamics of an avian scavenger guild that has been feeding on a mix of livestock and wildlife over a long historical period. The comparison of the hypothetical population growth as a result of animal biomass provided by the carcasses of wild ungulates alone with data obtained during censuses (with the availability of both wild and domestic carcasses plus food provided by feeding stations) allow us to discuss objectively the importance of wild ungulates as a food resource for the future management and conservation of avian scavengers. In parallel, we can also anticipate the effects that the hypothetical reduction or absence of carrion provided by domestic ungulates could have on a guild of avian scavengers. The dependence of vultures on extensive livestock on Mediterranean areas in which wild ungulate populations are not very important (e.g. Portugal, Greece, Sardinia, Corsica, [13]), allows our results to be extrapolated to these areas. As a result, we can generalize and discuss the possible effects that food availability limited to wild ungulates can have on vulture population dynamics in a Mediterranean context. Along these lines, in a hypothetical scenario in which restrictive public health policies could limit the availability of feeding resources provided by domestic carcasses [14], [17] , the impact that public health legislation may have on population growth allows us to discuss the relative importance of supplementary feeding and their management.

Our results suggest that there are significant differences between the two subpopulation areas, highlighting the importance of wild ungulates in one of them (the Pyrenees) in which population growth with wild ungulates alone being available is similar to the real population trend found in a scenario in which all food resources are available. The Spanish Pyrenean scenario is probably comparable to other European mountain areas as the Alps and the French Pyrenees in which wild ungulate populations are still important. Of the species present in the study area, in the Pyrenees it seems that primarily the Pyrenean chamois, and to a lesser extent the red deer, are two most important species as far as animal biomass provided is concerned. Thus, carrion provided by natural mortality or hunting practices (i.e. hunting trophy) involving these species is an important food resource for obligate scavengers [18], [20], [21] and also for facultative avian scavengers [29]. In addition, the carcasses can also been used by other species such as arthropods, bacteria and decomposing fungus [30].

In contrast, in the Pre-Pyrenees wild boar followed by red deer are the species that most animal biomass provided. However, in this area the food provided by wild ungulates alone seems insufficient to guarantee population growth. Thus, in this subpopulation there is a need for alternative food resources, such as those provided by domestic ungulates, to guarantee population growth. If densities of wild and domestic ungulates are insufficient, another possible means of maintaining avian scavenger guilds is the application of supplementary feeding activities. However, in this case their establishment should take into account the detrimental effects on both geographical expansion and fecundity [31]–[34]. For this purpose, and in order to optimise their management, the design of supplementary feeding sites should be based on studies that analyse inter- and intraspecific interactions, scavenging service efficiency and food preferences [35]–[37].

An important question is whether the availability of carrion provided by wild ungulates implies an important dilemma for managers. On the one hand, our results reveal the importance of wild carcasses for the viability of vulture populations. On the other hand, the detrimental effects of some carcasses, which were provided by hunting practices, due to lead poisoning need to be taken into account [11], [12], [38]. Thus, managers need answers in order to manage this resource, weighing up the pros and contras so as to optimise the management actions from a conservation point of view. Along these lines, the optimal solution is to avoid the disposal of carcasses provided by hunting practices with lead ammunition in order to minimise the risk of lead poisoning.

Our results suggests that it seems that whilst wild ungulates constitute an important food resource in the Pyrenees, they are insufficient to cover the energy requirements of avian scavenger guilds in the Pre-Pyrenees. These results could be extended to the rest of the southern chain of the Pyrenees and Pre-Pyrenees because, whilst wild ungulate populations are mainly found in the axial zone, higher densities of avian scavengers are found in the Pre-Pyrenees. In addition, these results also can be extrapolated to other Mediterranean scenarios such as Portugal, Italy, France or Greece, sharing similar ecological and socio-economic conditions. Along these lines, as our results suggest, public health legislation can modify population trends if a large number of domestic ungulate carcasses disappear from some mountain ranges [13]. In other endangered bearded vulture populations such as those of Corsica and Crete in which the dependence of the species on extensive grazing has also been suggested [39], [40], conservation concerns could be tested by applying similar procedures to optimize the application of conservation actions. In these cases, as our results suggest for the Pre-Pyrenees, supplementary feeding could be necessary if other alternative food resources are not available (i.e. the reintroduction of wild ungulates). In accordance with our results, feeding stations should be created, preferably in areas where there are low densities of wild ungulates. With regard to the animal biomass that wild ungulates can offer, taking into account the lead poisoning problem, priority actions should be the regulation of ammunition to avoid indirect poisoning of scavengers [41]. As a parallel management measure, it should by priority to eliminate the supply of carcasses of hunted animals to supplementary feeding sites. Moreover, in a scenario with a possible reduction in food resources as a result of public health legislation, the increase in the use of illegal poisoning [7], [8] and the presence of veterinary drugs [1], [2], [5] can also increase the problem of non-natural mortality, which affects population growth, especially in endangered species such as the bearded vulture in the Pyrenees [42]. Managers should anticipate the conservation actions required by assessing food availability and the possible scenarios in order to make the most suitable decisions. Approaches such as this study constitute a useful tool for attaining this objective.

## Materials and Methods

### Study area

The study was carried out in Catalonia (NE Spain). Three breeding vulture species (the bearded vulture, the Egyptian vulture and the griffon vulture) inhabit the study area. In addition, the six wild ungulates inhabiting the study area are the Pyrenean chamois, the red dee*r*, the fallow deer, the roe deer, the wild boar and the mouflon. Prey species are herbivores and their remains, together with those of domestic ungulates (sheep, goats, cattle, horses), form the basic source of nourishment for the avian scavengers in the study area (>80% of the diet is based on these species, [17], [43]).

The study area is characterised by the presence of two differentiated subpopulations, the Pyrenean region (57,244 km^2^) and the Pre-Pyrenean region (77,372 km^2^) which are interconnected. With regard to the distribution of the scavenger species, as a consequence of orography (i.e. more availability of suitable nesting sites) and weather (as a limiting factor for griffons and Egyptian vultures) the bearded vulture population in the Pyrenees *vs* the Pre-Pyrenees is 37.8% *vs* 62.2% (*n* = 37 pairs), the Egyptian vulture population 10.2% *vs* 89.8% (*n* = 59 pairs) and the griffon vulture 21.5% *vs* 78.5% (*n* = 822 pairs). Since individuals can move from one area to another depending on the resources available, the ecosystem appears to function as a single set (overall ecosystem) composed of two subsets (subpopulations separated by bio-geographical criteria). In this way, whenever there is a lack of trophic resources in one of the subareas, the individuals can move to the other one. In both, the ecosystem's local carrying capacity has been limited to the appropriate areas and habitats for the different species as well as has the maximum density that can be reached (Text S1, Table S1 and Table S2).

The study area contains a total of 15 feeding stations (four in the Pyrenees and 11 in the Pre-Pyrenees) in which food (principally bone remains) is provided between November-April principally for bearded vultures.

### Censuses and demographic parameters

Data on the avian scavenger and wild ungulate populations were obtained through censuses carried out by technicians from the Departament de Medi Ambient i Habitatge of the Generalitat de Catalunya, literature and personal observations from 1994–2008 [44], [45]. For each species we obtained parameters on breeding, energy requirements, mortality and the biomass that dead wild ungulates provided in the field, separating bone and meat remains in accordance with the different dietary habits between species (i.e. the diet of bearded vulture is based on bone remains whereas in the case of griffons and Egyptian vultures is based on meat [17], [43], [46]. Available grass biomass is enough to cover the energetic requirements of wild and domestic ungulates [47]–[50] and has not been considered as a limiting factor for the population dynamics of these species.

### Model used

A P-System is a computational model based on the structure of biological cells, and introduced by the mathematic researcher, Gheorghe Păun [22], [50]. Many of the studies that are carried out in the field of cell computation are developed not only using theoretical approaches, but also the modelling of real ecosystems [23], [24], [27], [51], [52].

**Figure 4:**
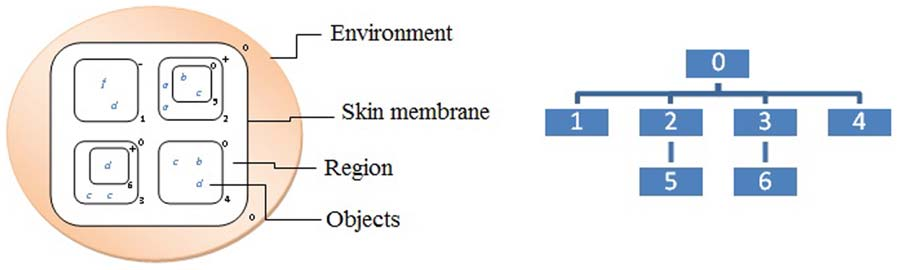
Graphic representation of a P-system formed by the membranes, regions and objects. The P-System is in an environment. At right the hierarchical representation of this structure of membranes.

Imitating the functioning and basic structure of cells, these models are made up of a membrane structure with a skin or external membrane that contains a set of other membranes, which separate the different parts of some cells. In order to differentiate the membranes, they are labelled numerically (subscript) and the membranes can have associated electrical charges (+, −, 0) (superscript). The structure of the membranes is represented using mathematical symbols 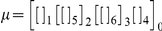 (Figure 4) or depicted graphically using a hierarchical tree (Figure 4). In the areas between the membranes there are sets of objects that are associated with the organelles that are found inside cells. Depending on the state of the environment in which they are located, these organelles evolve in different ways. The transformation that the objects undergo in the context of a P-system is represented through evolution rules. Each time a rule or a set or rules are applied, the configuration of the P-system changes. The main components of a P-system are: a membrane structure, a multi-set of objects and an evolution rule. The membrane structure allows us to separate processes that can work in parallel, increasing the efficiency of the model. The membrane structure is not limiting, but rather allows us to separate independent processes from sequence-dependent processes.

Each cell in an organism has its own life, although the cells communicate with one another. Therefore, different P-systems must be able to exchange information with each other. This is carried out using environments.

There are different types of P-systems, with different types of rules. In order to model processes related to population dynamics, we use the P-system known as the *Multienvironment probabilistic P-system with active membranes of degree (m, q)*, which is formally defined as:
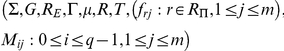
it can be viewed as a set of 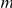 environments 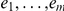 linked by the arcs from the directed graph 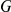. 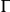 is the work alphabet (a set made up of all the objects that appear in the different configurations), 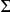 is an alphabet that represents the objects that can be present in the different environments. 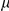 is the membrane structure and 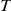 is the time.


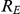
 are the rules that allow the communication between environments, they are of the form 
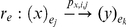
, and for each *x* it verifies 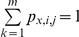. When a rule of this type is applied, the object 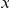 moves from environment 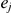 to environment 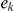 converted into 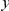, according to the probability 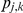.


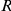
 are the rules that apply in the region of the P system, they are of the form: 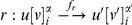 This rule applies with probability 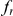 in the membrane labelled with 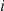, and with 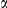 as an electrical charge if multiset 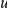 is contained in the membrane immediately outside of membrane *i* it is to said membrane father of membrane 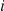 and multiset 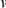 is contained in the membrane labelled with 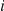 having 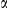 as an electrical charge. When that rule is applied, multiset 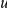 (respectively 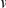) in the father of membrane 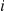 (respectively in membrane 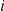) is removed from that membrane, and multiset 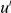 (respectively 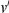) is produced in that membrane, changing its electrical charge to 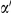.

The tuple 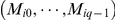 is the initial *configuration* of the P-system, that is to say that the objects there are in the regions in the initial moment in each environment *i*.

At some point, all the rules that can possibly be applied are applied, causing the P system to evolve and its configuration to change. A P system computation is made up of a sequence of configurations, which are obtained from previous computations through transitions.

The modularity of the P systems allows the problem to be broken down into modules, which can be applied sequentially or in parallel, and can also interrelate.

**Figure 5:**
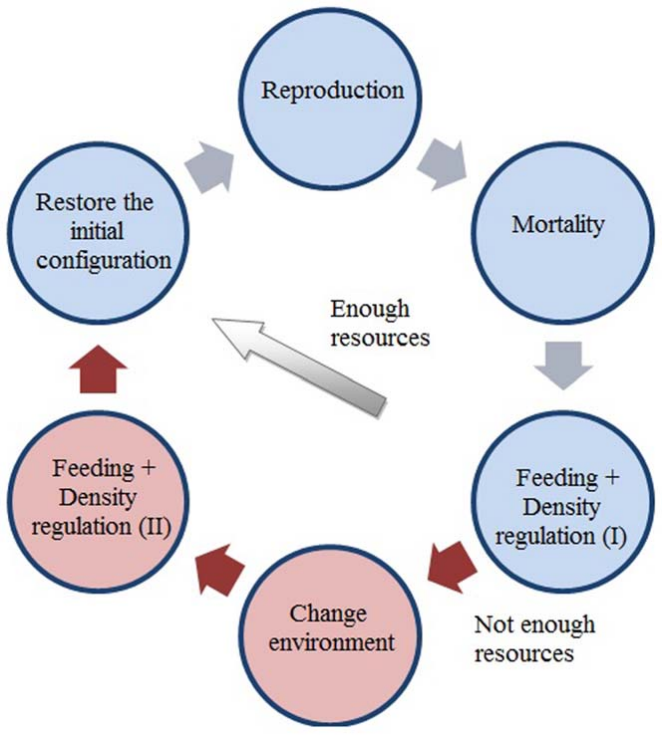
Sequence of the different modules that make up the model. The execution of a loop is equivalent to the passage of one year in the ecosystem.

The biological processes involved in the model used are: a) reproduction; b) mortality; natural or as a result of hunting (the possibility of the hunted animal being removed from the field or left in the field is also considered); c) feeding; d) control of the demographic load or density of each of the zones, and e) the possibility of the movement of the species between the two existing environments (Pyrenees and Pre-Pyrenees). The sequencing of the processes and modules that make up the model are shown in Figure 5.

The model used was executed using MeCoSim software (free software under licence), developed by members of the Natural Computation Group at the University of Sevilla (GNU GPL (http://www.p-lingua.org). The output that the simulator provides is the number of individuals of each species over the years simulated and the biomass generated in the form of bones and meat each year by each species, expressed in megacalories. These models' most important property is their capacity to work in parallel and to capture the randomness of the natural environmental processes using stochastic strategies based on Gillespie's theory of stochastic kinetics [53] and the semantics defined using probabilistic functions [27].

The model used is a *Multienvironment probabilistic P-system with active membrane of degree (2,2)*, two membranes and two environments. The membrane structure is defined in accordance with the problem to be solved and the strategy chosen by the model's designer, in this case has been 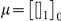. The initial configuration is 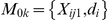, 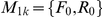 (k = 2 environments) each animal is associated with an object 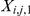 (except for the scavenger species in which a territory or colony is formed by a pair or several pairs), *i* is the species, *j* the age and 1 stands for the first year of simulation; this object and object 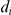 (*i* is the species) are found in the skin membrane labelled 0. In the inner membrane, labelled with a value of 1, are the objects 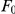 and 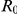; the first allows you to create objects associated with trophic resources provided by the environment, whilst the second object is used to synchronise the model.

In the *reproduction module* the objects 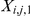 can evolve in different ways depending on the age (index *j*) and the sex. Moreover, not all females of fertile age breed each year. At the end of the reproduction module, the objects associated with the animals have evolved to objects of the type 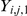 and appear new objects 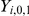.

In this same simulation step, the object 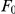 evolves creating objects associated with food provided in the supplementary feeding or generated by the ecosystem, in the case of the grass.

In the next step, the rules corresponding to the *mortality module* are applied. In this case the objects are not transformed if the animal survives but come into the inner membrane. In the other case they leave in the inner membrane objects associated with bones and meat that can be used as food for scavengers. Once the module has been completed, the objects associated with animals continue to be of type 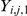, although at first they were in the skin membrane and are now in the inner membrane of the P system, which is where objects associated with food are found.

In order to synchronize the P system it is necessary to transform all the objects 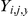 to 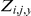. In the *feeding and density control module I* the object 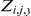 evolves to object 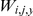 if there are resources and space if not; if it does not evolve, object 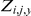 is sent to the environment and the *environment module* is applied.

After changing the environment, the *feeding and density control module II* rules are applied again. In this case, if there are no resources, the object associated with the animal disappears, evolving to biomass.

At this point, the rules needed to re-establish the initial configuration are applied. As a result of the application of the *module for restore the initial configuration*, the objects associated with animals are of type 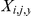 and the remaining objects recover the initial value, and the objects created during the different configurations are eliminated.

In order to apply the model, the parameters required (Table S1 and Table S2) were obtained from the literature and the authors' data [[27] and see details in Text S1].

Since several demographic parameters included in the model are variable and can influence the population dynamics, we assessed the elasticity of the model's response when we varied the selective parameters at the same level. We assessed the elasticity of the following parameters: m1, m2 (related with survival) k1 and k2 (related with reproductive parameters) (see Table 1). For this purpose, we used an interval range of these parameters according to values obtained from the literature (see Supporting information) and the authors' data in the study area. Using the definition of elasticity, we calculated the relative increase of the population according to the relative variation of these parameters by simulations as a consequence of the type of model used [25], [26].

### Scenarios analysed

We first compared the biomass provided by the different species in accordance with their spatial distribution (Pyrenees *vs* Pre-Pyrenees), in order to detect geographic differences in food availability. Next, for each region we determined the annual animal biomass provided by wild ungulates. Finally, we simulated the population trend taking into account the hypothetical growth with the availability of biomass provided by wild ungulates only in comparison with the real population growth obtained through censuses in which food available was provided by both wild and domestic ungulates and feeding stations. Data used for the posterior statistical analyses were obtained from the application of the model that simulates the population dynamics (10 years and a total of 100 replications) in a probabilistic way according to the randomness behaviour inherent to the problem.

### Statistical analyses

After the verification of normality (test of Kolmogorov-Smirnov) and homoscedasticity (Levene's test), we used one-way analysis of variance (ANOVA) to compare the biomass provided by the different species, taking into account the animal biomass as a dependent variable and the species as the factor. Where results from ANOVA were significant, a further homogeneity test using the Duncan test (*P*<0.05) was performed to identify inter-group differences. We used a *t*-test to compare the differences between the biomass provided by wild ungulates in each region (Pyrenees *vs* Pre-Pyrenees) and the percentage (log transformed) of annual population growth obtained with the models comparing the hypothetical population dynamics with the biomass provided by wild ungulates only to the real population growth.

## Supporting Information

Text S1 Supporting information.
(DOC)

Table S1 Values of parameters used in the model for each species. (F = female, M = male, A = spend the entire year in the mountain, P = spend part of the year in the ecosystem).
(DOCX)

Table S2Probability that the species moves between environments. 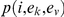: Probability that species 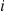 will move from environment 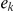 to environment 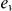 when there is a lack of resources. e_1_: Pyrenees; e_2_: Pre-Pyrenees.(DOCX)
