# Supplementary material for: Can Wild Ungulate Carcasses Provide Enough Biomass to Maintain Avian Scavenger Populations? An Empirical Assessment Using a Bio-Inspired Computational Model
Source: PLoS One. 2011 May 24;6(5):e20248. doi: 10.1371/journal.pone.0020248 (PMC3101228; doi:10.1371/journal.pone.0020248)
Supplement: Text S1 — Supporting information. (DOC) [file pone.0020248.s001.doc]

SUPPORTING INFORMATION

The ecosystem modelling is composed of two environments (Pyrenees and Pre-Pyrenees) and 18 types of animals. The number of animals per each species and environment as well as the number of years to be simulated are the input of the model.

The output is formed by the number of animals of each species for year and the biomass in megacalories that every species provides throughout the years simulated.

In order to model this ecosystem we use a *multienvironment probabilistic functional extended P system* with active membranes of degree (2, 2) (two membranes and two environments) taking $T$ time units (simulation years).

$$\left(\Sigma,G,R_{E},\Gamma,\mu,R,T\{f_{rj}:r\in R_{\Pi},1\leq j\leq2\},M_{ij}:0\leq i\leq1, 1\leq j\leq2\right)$$

The polarization of the membranes are used to show environmental changes (i.e. time of the year).

1. The membrane structure is$\mu=[[ ]_{1}]_{0}$
2. The initial configuration is $M_{0}=\left\{ X_{_{i,j,1}}^{q_{i,j}},d_{i} \right\}$ and$M_{1}=\{ R_{0},F_{0} \}$
3. The working alphabet of the P system is

$$\begin{matrix} \Gamma=\left\{ X_{i,j,y},Y_{i,j,y},Z_{i,j,y},Z'_{i,j,y},W_{i,j,y}:1\leq i\leq n, 0\leq j\leq g_{i,6}, 1\leq y\leq T \right\} \cup\\ \left\{ B,M,S,G,F_{0},F,b \right\} \cup\left\{ R_{i}:0\leq i\leq8 \right\} \cup\left\{ H_{i},C_{i},D_{i},d_{i},a_{i},e_{i}:1\leq i\leq n \right\} \end{matrix}$$

Objects $X_{i,j,y},Y_{i,j,y},Z_{i,j,y},Z'_{i,j,y}$ and $W_{i,j,y}$ represent the same animal but in different states. Index $i$ is associated with the type of animal, index $j$ is associated with the age (and $g_{i,6}$ is the average life expectancy) and $y$ is the simulation year. Objects$B$ and $H$represent bones, $C_{i}$ and $M_{i}$represent meat corresponding to the species $i$, $S$ represents meat from small animals and $G$is the amount of grass available for consumption in the ecosystem, $F_{0}$ and $F$ are used to generate external contributions. $D_{i}$ is an object used to count the existing animals of species $i$. If a species overcomes the maximum density values, it will be regulated. Objects $d_{i}$,$a_{i}$ and $e_{i}$ allow us to control the maximum number of animals per species in the ecosystem. When a regulation takes place, object$a_{i}$ allows us to eliminate the number of animals of species $i$ that exceeds the maximum density. $b$is an object used to change the charge of the membrane. At the end, object $R_{i}$ is a counter that allows the synchronization of the P system.

1. Environment alphabet is $\Sigma=\left\{ Z_{i,j,y},Z'_{i,j,y}:1\leq i\leq n, 1\leq j\leq g_{i,6}, 1\leq y\leq T \right\}$

Objects $Z_{i,j,y}$ and $Z'_{i,j,y}$ are associated with animals.

1. Rules $R$ and $R_{E}$ of the model.

The definitions of parameters that appear in the rules are:

$g_{i1}$: 1 wild animal and 0 domestic animals.

$g_{i2}$: proportion of time that animals of species *i* remain in the mountains during the year.

$g_{i3}$: age at which adult size is reached. This is the age at which the animal of species *i* consumes an adult diet with the same energetic requirements, and at which, if the animal dies, the amount of biomass it leaves is similar to the total left by an adult. Moreover, at this age it will have surpassed the critical early phase during which the mortality rate is high.

$g_{i4}$: age at which species *i* fertility begins.

$g_{i5}$: age at which species *i* fertility ends.

$g_{i6}$: average life expectancy of species *i* in the ecosystem.

$g_{i7}$: 1 if an important proportion of the diet of the species *i* can be based on other small species (i.e. carnivora, leporidae) and 0 for the remainder.

$k_{i1}$: in the case of ungulates, percentage of females of the species *i* presents in the population. For the scavengers, percentage of pairs of the species *i* that can breed. For both, scavengers and ungulates the sex-ratio at birth has been considered as 1:1.

$k_{i2}$: fertility ratio: proportion of fertile females that reproduce in the case of ungulates and proportion of pairs with successful breeding in the case of scavengers.

$k_{i3}$: number of descendants for fertile females of species *i* that reproduce.

$m_{i1}$: natural mortality ratio in first years of species *i*, age $<g_{i4}$ (per one).

$m_{i2}$: mortality ratio in adult animals of species *i*, age $\geq g_{i4}$(per one).

$m_{i3}$: is equal to 1 if the animal of the species *i* dies in the ecosystem and is not removed, and is equal to 0 if the animal is removed from the ecosystem before to die.

$f_{i1}$: amount of bones from young animals of species *i*, age $<g_{i4}$.

$f_{i2}$: amount of meat from young animals of species *i*, age $<g_{i4}$ .

$f_{i3}$: amount of bones from adult animals of species *i*, age $\geq g_{i4}$.

$f_{i4}$: amount of meat from adult animals of species *i*, age $\geq g_{i4}$.

$f_{i5}$: amount of bones necessary per year and pair (kg) of the species *i* according to the energetic requirements of the scavenger species.

$f_{i6}$: amount of grass necessary per year and animal of species *i* (kg).

$f_{i7}$: amount of meat necessary per year and pair (kg) animal of the species *i* according to the energetic requirements of the scavenger species.

$f_{i8}$: Percentage of useful bones left by species *i.*

$f_{i9}$: Percentage of useful meat left by species *i.*

*h_i_*_1_*:* percentage of young animals of the species *i* hunted.

*h_i_*_2_: percentage of adult animals of the species *i* hunted.

*h_i_*_3_: after hunting the body of the animal of the species *i* remain in the ecosystem (1) otherwise (0).

$d_{i1}$: maximum density of species *i* in the ecosystem.

$p_{ikv}$: probability that species $i$ will move from environment $k$ to environment $v$ when there is a lack of resources.

The values of the parameters for each species are shown in Tables 2 and 3.

In the *reproduction module* the objects$X_{i,j,1}$ can evolve in different ways depending on the age (index *j*) and the sex. Moreover, not all females of fertile age breed each year. In all cases, the rules that are applied are of type:

*k_i3_* is the number of offspring, with a value of 0 in the case of males, animals not of breeding age, and females that are of fertile age but do not breed.

At the end of the breeding module, the objects associated with the animals are of type $Y_{i,j,1}$

In this same simulation step, the following rules are applied $r\equiv\left(\left[F_{0}\xrightarrow{}B^{\alpha\left(k\right)}M^{\beta\left(k\right)}S^{\lambda\left(k\right)}G^{\gamma\left(k\right)}F\right]_{ 1}^{ 0}\right)_{e_{k}}$

$$r\equiv\left( d_{i}[ ]_{1}^{0}\xrightarrow{}\left[d_{i}a_{i}^{0,9\cdot d_{k,i,1}}e_{i}^{0,2\cdot d_{k,i,1}}\right]_{ 1}^{ 0} \right)_{ e_{k}}$$

$\alpha(k), \beta(k), \lambda(k)$ and $\gamma(k)$ are the amount of bones, meat, carcasses of small animals and grass provided externally in the environment *k*.

The first of these two rules will allow objects associated with the trophic resources available to be generated, whilst the second generates objects *a* and *e*, which allow the density of each species to be controlled.

In the next step, the rules corresponding to the *mortality module* are applied. They are rules of the following type

If the animals survive, rules of the first type are applied, and if they die rules of the second type are applied (they leave bones and meat biomass in the field, which can provide food for other species).

Once the module has been completed, the objects associated with animals continue to be of type $Y_{i,j,1}$, although at first they were in the skin membrane and are now in the inner membrane of the P system, which is where objects associated with food are found.

In the next step, and to ensure the model is consistent, it is necessary to transform all the objects $Y_{i,j,y}$ to $Z_{i,j,y}$. In the *feeding and density control module* the following type of rule is applied

$$r\equiv\left[Z_{i,j,y}a_{i}B^{f_{i,5}}G^{f_{i,6}}M^{f_{i,7}}S^{f_{i,8}}\right]_{ 1}^{ +}\xrightarrow{}[W_{i,j,y}]_{1}^{0}$$

If there are resources and space, object $Z_{i,j,y}$ evolves to object $W_{i,j,y}$; if not, it will not evolve; in this case the object $Z_{i,j,y}$is sent to the environment

$$r\equiv[Z_{i,j,y}]_{1}^{0}\xrightarrow{}Z_{i,j,y}[ ]_{1}^{0}$$

$$r\equiv[Z_{i,j,y}]_{0}^{0}\xrightarrow{}Z_{i,j,y}[ ]_{0}^{0}$$

From this environment, the object can reach the environment belonging to another area in which there may be trophic resources and space.

After changing the environment, the feeding and density control module rules are applied again. In this case, if there are no resources, the object associated with the animal disappears, evolving to biomass.

At this point, the rules needed to re-establish the initial configuration are applied. These are:

$$r\equiv[W_{i,j,y}]_{1}^{-}\xrightarrow{}X_{i,j+1,y+1}[ ]_{1}^{0}$$

$$r\equiv[R_{8}]_{1}^{-}\xrightarrow{}[R_{0}]_{1}^{0}$$

$$r\equiv[F]_{1}^{-}\xrightarrow{}[F_{0} ]_{1}^{0}$$

$$r\equiv[G]_{1}^{-}\xrightarrow{}[ \#]_{1}^{0}$$

$$r\equiv[M]_{1}^{-}\xrightarrow{}[ \#]_{1}^{0}$$

$$r\equiv[S]_{1}^{-}\xrightarrow{}[ \#]_{1}^{0}$$

As a result, the objects associated with animals are of type $X_{i,j,y}$ and the remaining objects recover the initial value, and the objects created during the different configurations are eliminated.

**Table legends**

Table S1. Values of parameters used in the model for each species. (F= female, M= male, A = spend the entire year in the mountain, P = spend part of the year in the ecosystem).

Table S2. Probability that the species moves between environments. $p\left(i,e_{k},e_{v}\right)$: Probability that species $i$ will move from environment $e_{k}$ to environment $e_{v}$ when there is a lack of resources. e_1_: Pyrenees; e_2_: Pre-Pyrenees.
