# Supplementary material for: Can Wild Ungulate Carcasses Provide Enough Biomass to Maintain Avian Scavenger Populations? An Empirical Assessment Using a Bio-Inspired Computational Model
Source: PLoS One. 2011 May 24;6(5):e20248. doi: 10.1371/journal.pone.0020248 (PMC3101228; doi:10.1371/journal.pone.0020248)
Supplement: Table S1 — Values of parameters used in the model for each species. (F = female, M = male, A = spend the entire year in the mountain, P = spend part of the year in the ecosystem). (DOCX) [file pone.0020248.s002.docx]

Table S1.

|  | g1 | g2 | g3 | g4 | g5 | g6 | g7 | k1 | k2 | k3 | m1 | m2 | m3 | f1 | f2 | f3 | f4 | f5 | f6 | f7 | f8 | f9 | h1 | h2 | h3 | d1 |
| --- | --- | --- | --- | --- | --- | --- | --- | --- | --- | --- | --- | --- | --- | --- | --- | --- | --- | --- | --- | --- | --- | --- | --- | --- | --- | --- |
| *Gypaetus barbatus^a^* | 1 | 1 | 1 | 6 | 20 | 21 | 0 | 0.65 | 0.35 | 1 | 0.06 | 0.08 | 1 | 0 | 0 | 0 | 0 | 135 | 0 | 45 | 0 | 0 | 0 | 0 | 0 | 119 |
| *Neophron percnopterus^b^* | 1 | 0.5 | 1 | 5 | 24 | 25 | 1 | 0.75 | 0.57 | 1 | 0.28 | 0.08 | 1 | 0 | 0 | 0 | 0 | 0 | 0 | 100 | 0 | 0 | 0 | 0 | 0 | 159 |
| *Gyps fulvus^c^* | 1 | 1 | 1 | 5 | 24 | 25 | 0 | 0.7 | 0.55 | 1 | 0.06 | 0.07 | 1 | 0 | 0 | 0 | 0 | 0 | 0 | 230 | 0 | 0 | 0 | 0 | 0 | 1399 |
| *Rupicapra pyrenaica^d^* | 1 | 1 | 1 | 2 | 18 | 18 | 0 | 0.55 | 0.75 | 1 | 0.6 | 0.06 | 1 | 3 | 4 | 6 | 24 | 0 | 550 | 0 | 0.5 | 0.5 | 0 | 3.5 | 0.25 | 14999 |
| *Cervus elaphus* (F)^e^ | 1 | 1 | 1 | 2 | 17 | 17 | 0 | 1 | 0.75 | 1 | 0.34 | 0.06 | 1 | 7 | 13 | 15 | 60 | 0 | 2540 | 0 | 0.6 | 0.6 | 0 | 0 | 0 | 4614 |
| *Cervus elaphus* (M)^e^ | 1 | 1 | 1 | 2 | 20 | 20 | 0 | 0 | 0 | 0 | 0.34 | 0.06 | 1 | 12 | 15 | 24 | 96 | 0 | 2540 | 0 | 0.6 | 0.6 | 0 | 0.3 | 1 | 2884 |
| *Dama dama^f^* | 1 | 1 | 1 | 2 | 12 | 12 | 0 | 0.75 | 0.55 | 1 | 0.5 | 0.06 | 1 | 1 | 14 | 2 | 37 | 0 | 1100 | 0 | 0.25 | 0.25 | 0 | 0 | 0 | 2999 |
| *Capreolus capreolus^g^* | 1 | 1 | 1 | 1 | 10 | 10 | 0 | 0.67 | 1 | 1 | 0.58 | 0.06 | 1 | 1 | 4 | 1 | 19 | 0 | 600 | 0 | 0.25 | 0.25 | 0 | 0 | 0 | 14999 |
| *Ovis orientalis^h^* | 1 | 1 | 1 | 2 | 12 | 12 | 0 | 0.5 | 0.9 | 2 | 0.6 | 0.06 | 1 | 3 | 4 | 6 | 22 | 0 | 550 | 0 | 0.6 | 0.6 | 0 | 0 | 0 | 999 |
| *Sus scrofa^j^* | 1 | 1 | 1 | 1 | 4 | 6 | 0 | 0.5 | 0.55 | 4 | 0.14 | 0.1 | 1 | 4 | 6 | 12 | 60 | 0 | 730 | 0 | 0.25 | 0.25 | 0.32 | 0.4 | 0 | 199999 |
| *Ovis aries* (A)^j^ | 0 | 1 | 1 | 2 | 8 | 8 | 0 | 0.96 | 0.75 | 1 | 0.15 | 0.03 | 0 | 3 | 4 | 7 | 38 | 0 | 1320 | 0 | 0.7 | 0.7 | 0 | 0 | 0 | 49999 |
| *Ovies aries* (P)^j^ | 0 | 0.5 | 1 | 2 | 8 | 8 | 0 | 0.96 | 0.75 | 1 | 0.15 | 0.03 | 0 | 3 | 4 | 7 | 38 | 0 | 1320 | 0 | 0.7 | 0.7 | 0 | 0 | 0 | 49999 |
| *Bos taurus* (A)^k^ | 0 | 1 | 2 | 2 | 9 | 14 | 0 | 0.9 | 0.9 | 1 | 0.057 | 0.045 | 0 | 10 | 60 | 6 | 518 | 0 | 11000 | 0 | 0.6 | 0.6 | 0 | 0 | 0 | 168499 |
| *Bos taurus* (P)^k^ | 0 | 0.4 | 2 | 2 | 9 | 14 | 0 | 0.9 | 0.9 | 1 | 0.057 | 0.045 | 0 | 10 | 60 | 6 | 518 | 0 | 11000 | 0 | 0.6 | 0.6 | 0 | 0 | 0 | 168501 |
| *Capra hircus* (A)^l^ | 0 | 1 | 1 | 2 | 8 | 8 | 0 | 0.97 | 0.9 | 1 | 0.12 | 0.015 | 0 | 3 | 4 | 9 | 37 | 0 | 1400 | 0 | 0.6 | 0.6 | 0 | 0 | 0 | 16999 |
| *Capra hircus* (P)^l^ | 0 | 0.5 | 1 | 2 | 8 | 8 | 0 | 0.97 | 0.9 | 1 | 0.12 | 0.015 | 0 | 3 | 4 | 9 | 37 | 0 | 1400 | 0 | 0.6 | 0.6 | 0 | 0 | 0 | 16999 |
| *Equus caballus* (A)^m^ | 0 | 1 | 3 | 3 | 9 | 20 | 0 | 0.97 | 0.9 | 1 | 0.034 | 0.0142 | 0 | 10 | 60 | 9 | 891 | 0 | 12000 | 0 | 0.8 | 0.8 | 0 | 0 | 0 | 6599 |
| *Equus caballus* (P)^m^ | 0 | 0.55 | 3 | 3 | 9 | 20 | 0 | 0.97 | 0.9 | 1 | 0.034 | 0.0142 | 0 | 10 | 60 | 9 | 891 | 0 | 12000 | 0 | 0.8 | 0.8 | 0 | 0 | 0 | 6599 |

$g_{1}$: 1 wild animal and 0 domestic animals.

$g_{2}$: proportion of time that animals remain in the mountains during the year.

$g_{3}$: age at which adult size is reached. This is the age at which the animal consumes an adult diet, and at which if the animal dies, the amount of biomass it leaves is similar to the total left by an adult. Moreover, at this age it will have surpassed the critical early phase during which the mortality rate is high.

$g_{4}$: age at which fertility begins.

$g_{5}$: age at which fertility ends.

$g_{6}$: average life expectancy in the ecosystem.

$g_{7}$: 1 if an important proportion of the diet of the species can be based on other small species (i.e. carnivora, leporidae) and 0 for the remainder.

$k_{1}$: in the case of ungulates, percentage of females presents in the population. For the scavengers, percentage of pairs that can breed. For both, scavengers and ungulates the sex-ratio at birth has been considered as 1:1.

$k_{2}$: fertility ratio: proportion of fertile females that reproduce in the case of ungulates and proportion of pairs with successful breeding in the case of scavengers.

$k_{3}$: number of descendants for fertile females that reproduce.

$m_{1}$: natural mortality ratio in first years, age $<g_{4}$ (per one).

$m_{2}$: mortality ratio in adult animals, age $\geq g_{4}$(per one).

$m_{3}$: is equal to 1 if the animal dies in the ecosystem and is not removed, and is equal to 0 if the animal is removed from the ecosystem before to die.

$f_{1}$: amount of bones (kg) from young animals, age $<g_{4}$.

$f_{2}$: amount of meat (kg) from young animals, age $<g_{4}$.

$f_{3}$: amount of bones from adult animals, age $\geq g_{4}$.

$f_{4}$: amount of meat (kg) from adult animals, age $\geq g_{4}$.

$f_{5}$: amount of bones necessary per year and pair (kg) according to the energetic requirements of the scavenger species (see references in a, b and c).

$f_{6}$: amount of grass necessary per year and animal (kg).

$f_{7}$: amount of meat necessary per year and pair (kg) according to the energetic requirements of the scavenger species (see references in a, b and c).

*f*_8_: percentage of useful bones according to personal observations and unpublished data.

*f_9_:* percentage of useful meat according to personal observations and unpublished data.

*h*_1_*:* percentage of young animals hunted.

*h*_2_: percentage of adult animals hunted.

*h*_3_: after hunting the body remain in the mountain (1) otherwise (0).

*d*_1_: maximum carrying capacity taking into account both subpopulations.

a.

Antor RJ, Margalida A, Frey H, Heredia R, Lorente L, Sesé JA (2007) Age of first breeding in wild and captive populations of Bearded Vultures (*Gypaetus barbatus*). Acta Ornithol 42: 114-118.

Donázar JA (1993) *Los buitres ibéricos: biología y conservación*. Madrid: JM Reyero Editor.

Donázar JA, Margalida A, Campión D (2009) *Vultures, feeding stations and sanitary*

*legislation: a conflict and its consequences from the perspective of conservation biology*. Munibe 29 (Suppl.). Donostia: Sociedad de Ciencias Aranzadi.

García D, Margalida A (2009) Status, distribution and breeding parameters of the

scavengers in Catalonia. In: Donázar JA, Margalida A, Campión D, editors. Vultures, feeding stations and sanitary legislation: a conflict and its consequences from the perspective of conservation biology. Munibe 29 (Suppl.). Donostia: Sociedad de Ciencias Aranzadi. 116-135.

Margalida A (2010) *Conservation biology of the last and largest natural population of the European bearded vulture* (Gypaetus barbatus) *(Linnaeus, 1758).* PhD thesis. Bern: University of Bern.

Oro D, Margalida A, Carrete M, Heredia R, Donázar JA (2008) Testing the goodness of supplementary feeding to enhance population viability in an endangered vulture. PLoS ONE 3: e4084.

b.

Donázar JA (1993) *Los buitres ibéricos: biología y conservación*. JM Reyero Editor, Madrid.

Donázar JA, Margalida A, Campión D (2009) *Vultures, feeding stations and sanitary*

*legislation: a conflict and its consequences from the perspective of conservation biology*. Munibe 29 (Suppl.). Donostia: Sociedad de Ciencias Aranzadi.

Grande JM (2006) Factores limitantes, antrópicos y naturales de poblaciones de aves carroñeras: el caso del alimoche (*Neoprhon percnopterus*) en el Valle del Ebro. PhD thesis. University of Sevilla, Sevilla.

Grande JM., Serrano D, Tavecchia G, Carrete M, Ceballos O et al. (2009) Survival in a long-lived territorial migrant: effects of life-history traits and ecological conditions in wintering and breeding areas. Oikos 118: 580-590.

c.

Blanco G, Martínez F, Traverso JM (1997) Pair bond and age distribution of breeding griffon vultures *Gyps fulvus* in relation to reproductive status and geographic area in Spain. Ibis 139: 180-183.

Donázar JA (1993) *Los buitres ibéricos: biología y conservación*. JM Reyero Editor, Madrid.

Donázar JA, Margalida A. Campión D (2009) Vultures, feeding stations and sanitary

legislation: a conflict and its consequences from the perspective of conservation biology. Munibe 29 (Suppl.). Donostia: Sociedad de Ciencias Aranzadi.

Le Gouar P, Robert A, Choisy JP, Henriquet S, Lecuyer P, Tessier Ch, Sarrazin F

(2008) Roles of survival and dispersal in reintroduction success of griffon vulture (*Gyps fulvus*). Ecol Appl 18: 859-872.

d.

García-González R, Herrero J, Hidalgo R (1985) Estimación puntual de diversos parámetros poblacionales y distributivos del sarrio en el Pirineo Occidental. Pirineos 35: 53-63.

Herrero J, Escudero E, Fernández de Luco D, García-González R (Eds) (2004) Biología, patología y gestión del sarrio. Zaragoza: Publicaciones del Consejo de Protección de la Naturaleza de Aragón, Serie Investigación, nº 46.

Pérez-Barbería FJ, García-González R, Palacios B (2010) Rebeco *Rupicapra pyrenaica*. In: Salvador A, Cassinello J, editors. Enciclopedia Virtual de los Vertebrados Españoles. Museo Nacional de Ciencias Naturales, Madrid http://www.vertebradosibericos.org/

e.

Bonenfant C, Gaillard J M, Klein F, Loison A, Klein F (2003) Sex-ratio variation and reproductive costs in relation to density in a forest-dwelling population of red deer (*Cervus elaphus*). Behav Ecol 14: 862-869.

Carranza J (2004) Ciervo Cervus *elaphus*. In: Carrascal LM, Salvador A, editors. Enciclopedia Virtual de los Vertebrados Españoles*.* Museo Nacional de Ciencias Naturales, Madrid. http://www.vertebradosibericos.org/

Carranza J, Martínez, JG, Sanchez-Prieto C, Fernández-García JL, Sánchez-Fernández B et al. (2003) Game species: extinctions hidden by census numbers. Anim Biodiv Conserv 26: 81-84.

Kruuk LE, Clutton-Brock TH, Albon SD, Pemberton JM, Guinness FE (1999) Population density affects sex ratio variation in red deer. Nature 399: 407-408.

Martínez M, Rodriguez-Vigal C, Jones OR, Coulson T, San Miguel A (2005) Different hunting strategies select for different weights in red deer. Biol Lett 1: 353-356.

Rose KE, Clutton-Brock TH, Guinness FE (1998) Cohort variation in male survival and lifetime breeding success in red deer. J Anim Ecol 67: 979-986.

Torres-Porras J, Carranza J, Pérez-González J (2009) Selective culling of Iberian red deer stags (*Cervus elaphus hispanicus*) by selective montería in Spain. Eur J Wildl Res 55: 117-123.

Yoccoz NG, Mysterud A, Langvatn R, Stenseth NC (2002) Age and density-dependent reproductive effort in male red deer. Proc R Soc Lond B 269: 1523-1529.

f.

Braza F (2003) Gamo Dama dama. In: Carrascal LM, Salvador A, editors. Enciclopedia Virtual de los Vertebrados Españoles. Museo Nacional de Ciencias Naturales, Madrid http://www.vertebradosibericos.org/

Braza F, San José C, Blom A (1988) Birth measurements, parturition dates, and progeny sex ratio of Dama dama in Doñana, Spain*.* J Mammal 69: 607-610.

Komers PE, Pélabon C, Stenström D (1997) Age at first reproduction in male fallow deer: age-specific versus dominance-specific behaviors. Behav Ecol 8: 456-462.

Say L, Naulty F, Hayden TJ (2003) Genetic and behavioural estimates of reproductive skew in male fallow deer. Mol Ecol 12: 2793-2800.

g.

Andersen R, Duncan P, Linnell JDC (1998) *The European roe deer: the biology of success*. Scandinavian University Press, Oslo.

Andersen R, Gaillard J-M, Linnell JDC, Duncan P (2000) Factors affecting maternal care in an income breeder the European roe deer. J Anim Ecol 69: 672-682.

Delibes JR (1996) *Ecología y comportamiento del corzo* (Capreolus capreolus *L. 1758*) *en la sierra de Grazalema (Cádiz).* PhD Thesis. University Complutense of Madrid, Madrid.

Hewison, AJM, Gaillard JM (1996) Birth-sex ratios and local resource competition in roe deer, *Capreolus capreolus*. Behav Ecol 7: 461 464.

# Mateos-Quesada P (2002) *Biología y comportamiento del corzo ibérico*. Servicio de Publicaciones Universidad de Extremadura, Cáceres.

# Mateos-Quesada P (2005) Corzo *Capreolus* *capreolus*. En: Enciclopedia Virtual de los Vertebrados Españoles. Carrascal LM, Salvador A (Eds). Museo Nacional de Ciencias Naturales, Madrid. http://www.vertebradosibericos.org/

# Mateos-Quesada P (2002) *Biología y comportamiento del Corzo Ibérico*. PhD Thesis. University of Extremadura, Cáceres.

Mateos-Quesada PY, Carranza J (2000) Reproductive patterns of roe deer in central

Spain. Etología 8: 9-12.

Telleria JL, Virgós E (1997) Distribution of an increasing roe deer population in a fragmented Mediterranean landscape. Ecography 20: 247-252.

h.

Bon R, Dardaillon M, Estévez I (1993) Mating and lambing periods as related to age of female mouflon. J Mammal 74: 752-757.

Bonenfant C, Pelletier F, Garel M, Bergeron P (2009) Age-dependent relationship between horn growth and survival in wild sheep. J Anim Ecol 78: 161-171.

Cassinello J (2003) Muflón *Ovis orientalis*. In: Carrascal LM, Salvador A, editors. Enciclopedia Virtual de los Vertebrados Españoles. Museo Nacional de Ciencias Naturales, Madrid. <http://www.vertebradosibericos.org/>

Faliu L, Cugnasse JM, Auvray F, Orliac D, Rech J (1990) Le régime alimentaire du mouﬂon de Corse (*Ovis ammon musimon*) dans le massif du Caroux Espinouse d’après l’analyse du contenu de 125 panses. Rev Med Vet 141: 545-556.

Garel M, Cugnasse JM, Maillard D, Gaillard JM, Hewison AJ et al. (2007) Selective harvesting and habitat loss produce long-term life history changes in a mouflon population. Ecol Appl 17: 1607-1618.

Garel M, Cugnasse JM. Gaillard A, Loison P, Gibert P et al. (2005). Reproductive output of female mouﬂon (*Ovis gmelini musimon* x *Ovis sp*.): a comparative analysis. J Zool 266: 65-71.

Santiago Moreno J, Toledano Díaz A, Gómez Brunet A, López Sebastián A (2004) El muflón europeo (*Ovis orientalis musimon* Schreber, 1782) en España: consideraciones históricas, filogenéticas y fisiología reproductiva. Galemys: 16: 3-20.

i.

Fernández-Llario P (2006) Jabalí – *Sus scrofa.* In: Carrascal LM, Salvador A, editors. Enciclopedia Virtual de los Vertebrados Españoles. Museo Nacional de Ciencias Naturales, Madrid. http://www.vertebradosibericos.org/

Herrero J, García-Serrano A, García-González R (2008) Reproductive and demographic parameters in two Iberian wild boar *Sus scrofa* populations. Acta Theriol 53: 355-364.

Sáez-Royuela C (1989) *Biología y ecología del jabalí*. PhD Thesis. University of Madrid, Madrid.

Rosell C (1998) *Biologia i ecologia del senglar* (Sus scrofa *L, 1758*) *a dues poblacions del nordest ibèric. Aplicació a la gestió*. PhD Thesis. University of Barcelona, Barcelona.

j.

# Avellanet R (2006) *Conservación de recursos genéticos ovinos en la raza Xisqueta: caracterización estructural, racial y gestión de la diversidad en programas “in situ”*. PhD Thesis. University Autònoma de Barcelona, Barcelona.

de Rancourta M, Foisb N, Lavínc MP, Tchakériand E, Vallerande F (2006)

Mediterranean sheep and goats production: An uncertain future. Small Ruminant Research 62: 167-179.

Roigé Ventura X (coord.) (1995) *Pirineo Catalán*. Cuadernos de la Trashumancia nº 13. Madrid: ICONA.

Oregui LM, Falagán A (2006) Spéciﬁcité et diversité des systèmes de production ovine

et caprine dans les Bassin Méditerranéen (Speciﬁcity and diversity of the sheep and goat systems in the Mediterranean basin). Options Méditerranéennes A-70:77–86.

k.

# Casellas J, Piedrafita J (2002) Correction factors for weight productive traits up to weaning in the Bruna dels Pirineus beef cattle breed. Anim Res 51: 43-50.

# Majoral D (1987) *El sector lleter a les comarques de muntanya. L'Alt Urgell*. Barcelona. Generalitat de Catalunya. DGPT.

# Serra X (2001) *La raça bovina Bruna dels Pirineus: qualitat de la canal i de la carn. Característiques bioquímiques del múscul* longissimus thoracis *en set races autòctones*. PhD Thesis. University Autònoma de Barcelona, Barcelona.

# Tarrés J (2006) *Estudi de la longevitat i la supervivència fins al deslletament en bovins de muntanya de la raça Bruna dels Pirineus*. PhD Thesis. University Autònoma de Barcelona, Barcelona.

l.

de Rancourta M, Foisb N, Lavínc MP, Tchakériand E, Vallerande F (2006)

Mediterranean sheep and goats production: An uncertain future. Small Rumiant Res 62: 167-179.

Departament d'Agricultura, Ramaderia, Pesca, Alimentació i Medi Natural (2010). Oví i

Cabrum, fitxa estadística sectorial. <http://www20.gencat.cat/docs/DAR/DE_Departament/DE02_Dades_estadistiques/05_Fitxes_sectorials/Sectors_Ramaders/Ovi%20%20cabrum/Documents/Fitxers_estatics/OVI_CABRUM.pdf>.

Oregui LM, Falagán A (2006) Spéciﬁcité et diversité des systèmes de production ovine

et caprine dans les Bassin Méditerranéen (Speciﬁcity and diversity of the sheep and goat systems in the Mediterranean basin). Options Méditerranéennes A-70:77-86.

Roigé Ventura X (1995) *Pirineo Catalán*. Cuadernos de la Trashumancia nº 13. ICONA, Madrid.

m.

Aldezabal A, Garin I, García-González R (1999) Activity rhythms and the influence of

some environmental variables on summer ungulate behaviour in Ordesa-Monte Perdido National Park. Pirineos 153-154:145-157.

Aldezabal A (1997) *Análisis de la interacción vegetación-grandes herbívoros en las*

*comunidades supraforestales del Parque Nacional de Ordesa y Monte Perdido (Pirineo Central, Aragón)*. PhD Thesis. University of País Vasco, Bilbao.

Cabús C (1988) La Cria del Cavall Hispa-Bretó de la Cerdanya. Revista de l’Institut

Agrícola Català de Sant Isidre. Hivern 87: 9-16.

Danell K, Bergstrom R, Duncan P, Pastor J (2006) *Large herbivore ecology,*

*ecosystem dynamics and conservation*. Cambridge Univiversity Press, Cambridge.

Derner JD, Hart RH, Smith MA, Waggoner JW (2008) Long-term cattle gain responses

to stocking rate and grazing systems in northern mixed-grass prairie. Livestock Science 117: 60-69.

Duncan P (1983) Determinats of the use of habitat by horses in a Mediterranean

wetland. J Anim Ecol 52: 93-109.

Fleurance G, Duncan P, Fritz H, Gordon IJ, Grenier-Loustalot MF (2010) Influence of

sward structure on daily intake and foraging behaviour by horses. Animal 4: 480-485.

Hart RH, Samuel MJ, Test PS, Smith MA (1988) Cattle, vegetation, and economic

responses to grazing systems and grazing pressure. J Range Manage 41:282-286.

Howery LD, Provenza FD, Banner RE, Scott CB (1998) Social and enviromental

factors influence cattle. Appl Anim Behav Science 55: 231-244.

Infante J (2008) *Caracterización Estructural de las Explotaciones Equinas de Carne del*

*Pirineo Catalán. Caracterización Morfológica de la Raza: Caballo Pirenaico Catalán*. University Autònoma de Barcelona, Barcelona.

Sagarra M (2007) *Situació actual i evolució del sector equí de carn als Pirineus*

*Catalans*. ETSEA. Universitat de Lleida, Lleida, <http://www.rac.uab.es/bibliografia/articles/AHP/TesinaMSM.pdf>
