# Supplementary material for: Can Wild Ungulate Carcasses Provide Enough Biomass to Maintain Avian Scavenger Populations? An Empirical Assessment Using a Bio-Inspired Computational Model
Source: PLoS One. 2011 May 24;6(5):e20248. doi: 10.1371/journal.pone.0020248 (PMC3101228; doi:10.1371/journal.pone.0020248)
Supplement: Table S2 — Probability that the species moves between environments. : Probability that species will move from environment to environment when there is a lack of resources. e1: Pyrenees; e2: Pre-Pyrenees. (DOCX) [file pone.0020248.s003.docx]

Table S2.

|  | $p\left(i,e_{1},e_{1}\right)$ | $p\left(i,e_{1},e_{2}\right)$ | $p\left(i,e_{2},e_{1}\right)$ | $p\left(i,e_{2},e_{2}\right)$ |
| --- | --- | --- | --- | --- |
| *Gypaetus barbatus* | 0 | 1 | 0 | 1 |
| *Neophron percnopterus* | 0 | 1 | 0 | 1 |
| *Gyps fulvus* | 0 | 1 | 0 | 1 |
| *Rupicapra pyrenaica* | 0.95 | 0.05 | 0.95 | 0.05 |
| *Cervus elaphus* (female) | 0.8 | 0.2 | 0.8 | 0.2 |
| *Cervus elaphus* (male) | 0.8 | 0.2 | 0.8 | 0.2 |
| *Dama dama* | 0.98 | 0.02 | 0,98 | 0.02 |
| *Capreolus capreolus* | 0.7 | 0.3 | 0.7 | 0.3 |
| *Ovis orientalis* | 0.995 | 0.005 | 0.995 | 0.005 |
| *Sus scrofa* | 0.98 | 0.02 | 0.98 | 0.02 |
| *Ovis aries* (adult) | 1 | 0 | 1 | 0 |
| *Ovis aries (*young) | 1 | 0 | 1 | 0 |
| *Bos taurus* (adult) | 1 | 0 | 1 | 0 |
| *Bos taurus* (young) | 1 | 0 | 1 | 0 |
| *Capra hircus* (adult) | 1 | 0 | 1 | 0 |
| *Capra hircus* (young) | 1 | 0 | 1 | 0 |
| *Equus caballus* (adult) | 1 | 0 | 1 | 0 |
| *Equus caballus* (young) | 1 | 0 | 1 | 0 |
